# Testosterone Replacement in Prostate Carcinoma: A Systematic Review of an Emerging Paradigm and Therapeutic Potential

**DOI:** 10.7759/cureus.94872

**Published:** 2025-10-18

**Authors:** Jared Robinson, Indrajit Banerjee, Indraneel Banerjee

**Affiliations:** 1 Surgery, Joe Morolong Memorial Hospital, Vryburg, ZAF; 2 Pharmacology, Sir Seewoosagur Ramgoolam Medical College, Belle Rive, MUS; 3 Urology, University of Florida Health, Jacksonville, USA

**Keywords:** exogenous testosterone use, hormone replacement therapy, onco-urology, prostate, prostatic neoplasms, testosterone (tt)

## Abstract

Prostate carcinoma is one of the most prevalent cancers among men. It is a dreaded outcome in the elderly male population and most commonly affects those 65 years of age and older. The etiologic nature of this common urological cancer is multifactorial and has an environmental, racial, and genetic interplay. One of the important factors believed to be involved in the causation of prostate carcinoma are androgens and prostatic sensitivity. Conventional treatment for this neoplasm ranges from radical surgery (prostatectomy), external beam radiation, brachytherapy, and androgen deprivation therapy (ADT) to radiopharmaceutical techniques. The conventional consensus is that external testosterone replacement therapy (TRT) is contraindicated in patients with prostate carcinoma or who are at risk thereof. The latest data and studies are challenging this notion and relationship. It is paramount that these misconceptions and older conventions are definitively and clearly corrected so as to ensure the best and most suitable treatment is made available to patients. This systematic review aims to find the relation between testosterone replacement therapy and prostate carcinoma.

An extensive review of literature was done on the following databases: Google Scholar, Trip Database, EMBASE, PubMed, and PubMed Central to collate the latest data available on testosterone replacement therapy and prostate carcinoma for a systematic review. A combination of keywords was used for data extraction: “Hormone Replacement Therapy” OR “Prostate” OR “Prostatic Neoplasms” OR “Testosterone” OR “Urology.”

A triad of findings were noted: (1) exogenous testosterone replacement therapy is not contraindicated in men who have undergone definite treatment for their prostatic carcinoma (prostatectomy, external beam radiation, and/or chemotherapy), (2) exogenous testosterone replacement therapy does not alter intraprostatic dehydroepiandrosterone (DHEA) levels and thus does not attenuate or catalyze any change in the prostatic milieu, and (3) castration-resistant prostatic carcinoma does not undergo disease progression when treated using high-dose testosterone replacement therapy.

No correlation between testosterone replacement therapy and prostate carcinoma exists, and TRT is not contraindicated in men post-definitive treatment for their primary neoplastic prostatic lesion. TRT is indicated in hypogonadal men post-primary treatment for its myriad of benefits and ultimate improvement of quality of life. The role of TRT in men prior to the diagnosis of prostatic carcinoma is unclear; thus, patients should give their informed consent before receiving the testosterone therapy.

The role of TRT in prostate cancer is evolving, even in cases of advanced prostate cancer. One area of interest is bipolar androgen therapy (BAT), which has been studied in various clinical trials focused on castrate-resistant prostate cancer. Prostate cancer cells adapt to chronic low testosterone levels by increasing androgen receptor activity, resulting in a 30- to 90-fold rise in androgen receptor levels. Although this significant upregulation of androgen receptors can lead to castration resistance, it also creates a therapeutic vulnerability to treatment with high doses of testosterone, which can result in growth arrest or cell death. The term “bipolar” in BAT refers to the rapid switching between two extremes: from high testosterone levels (supraphysiologic) to near-castrate serum testosterone levels.

## Introduction and background

Prostate carcinoma has forever been a burden for the male gender to bear. It has a dreaded outcome in the elderly male population and most commonly affects those aged 65 years and older [1]. According to Global Cancer Statistics 2022 (GLOBOCAN 2022) estimates, prostate carcinoma ranked fourth in global incidence, with an estimated 1,467,854 new cases reported in 2022, accounting for 7.34% of all new cancer cases that year [2]. Prostate carcinoma has a multifactorial etiology. The underlying cause of this common urological cancer has an environmental, racial, and genetic interplay. One of the important factors believed to be involved in the causation of prostate carcinoma is androgens and prostatic sensitivity [3,4]. Prostate carcinoma often follows an indolent course and is often asymptomatic or may present with minimal symptoms resembling its benign counterpart, benign prostatic hyperplasia (BPH), and therefore, patients may seek medical attention toward advanced stages of the disease, which makes treatment challenging. Conventional treatment for this neoplasm ranges from radical surgery (prostatectomy), external beam radiation, brachytherapy, and androgen deprivation therapy (ADT) to radiopharmaceutical techniques. The majority of which usually render the male hypogonadal, which innately has adverse effects of its own [5,6]. The conventional consensus that external testosterone replacement therapy (TRT) is contraindicated in patients with prostate carcinoma or who are at risk was based on the theory that androgens such as testosterone induce prostatic neoplastic events and are paramount in catalyzing such prostatic neoplasms. Many urologists have even viewed testosterone replacement therapy as an absolute contraindication, even in those men who have received definitive treatment for their prostate cancer. This relationship between the use of testosterone replacement therapy and prostate carcinoma is being challenged by newer studies, with a complete juxtaposition being displayed in the treatment of prostate carcinoma with the adjunctive use of TRT [7-9].

The use of TRT for patients with prostate cancer is supported by the "Prostate Saturation Model." This model suggests that prostate growth is highly sensitive to variations in androgen concentrations at low levels but becomes less sensitive to changes in androgen concentrations at higher levels. This pattern aligns with the observation that androgens exert their effects on the prostate primarily through binding to the androgen receptor, with maximal androgen-receptor binding occurring at serum testosterone concentrations significantly below the physiological range [10]. The validity of this model is further supported by an observational study published by Khera et al. in 2011 [11]. In this study, 451 hypogonadal men began TRT for 12 months and were divided into two groups based on their pre-TRT serum testosterone levels: group A (testosterone < 250 ng/dL) and group B (testosterone ≥ 250 ng/dL). After 12 months of TRT, a significant increase in serum prostate-specific antigen (PSA) levels was observed only in group A [11].

The misconceptions around the use of TRT and prostate cancer are all too common, and it is paramount that these misconceptions and older conventions are definitively and clearly corrected so as to ensure the best and most suitable treatment is made available to patients suffering from this life-threatening neoplasm. This systematic review aims to find the relation between TRT and prostate carcinoma and debunk the misconceptions surrounding TRT in prostate cancer.

## Review

Methodology

Preferred Reporting Items for Systematic Reviews and Meta-Analyses (PRISMA) guidelines 2020 were followed for this systematic review.

Literature Searches

An extensive review of literature was done on the following databases to collate the latest data available on testosterone replacement therapy and prostate carcinoma for this systematic review: Google Scholar, Trip Database, EMBASE, PubMed, and PubMed Central. A combination of keywords was used for data extraction: “Hormone Replacement Therapy” OR “Prostate” OR “Prostatic Neoplasms” OR “Testosterone” OR “Urology.”

Inclusion Criteria

All published full-text randomized controlled clinical trials in the English literature were included in the study. All randomized controlled clinical trials completed and published between January 1, 2000, and September 15, 2025, which focused on testosterone replacement therapy and prostate carcinoma, were independently and thoroughly screened by the three investigators (JR, IB, and IB) and were subsequently included in this study. A total of 341,839 articles from PubMed, 150,000 from Google Scholar, 6,722 from EMBASE, and 22,817 from the Trip Database were identified and included in this study, which accounts for a total of 521,378 articles. 

Exclusion Criteria

The choice to exclude a study was dependent on the availability of data concerning testosterone replacement therapy and prostate carcinoma. Non-randomized controlled trials (NRCTs), abstracts, cross-sectional studies, cohort studies, case series, case study, reports, editorial, viewpoint, and letter to the editor type of manuscripts were precluded from this study.

Data Extraction

Appropriate titles of the studies were initially searched for on the relevant databases. The selected titles were then screened; abstracts and full texts of randomized controlled trials, and those that met the eligibility requirements were considered for ultimate selection. All of the literature evaluation was independently conducted by the three researchers (JR, IB, and IB). The extracted data include study authors, year, gender, design, sample size, study population, control, biochemical profile, baseline PSA level, testosterone level, intraprostatic dehydroepiandrosterone (DHEA), drug combination, therapeutic protocol, findings, study limitations, and study outcomes.

Methodology Quality Assessment

For all the randomized controlled trials included in this systematic review, the Cochrane Risk of Bias Tool (RoB 2) was used. JR, IB, and IB independently made the quality assessment. Weighted bar plots and traffic light plots were generated for the risk of bias assessment data using the robvis visualization tool.

Results

**Figure 1:**
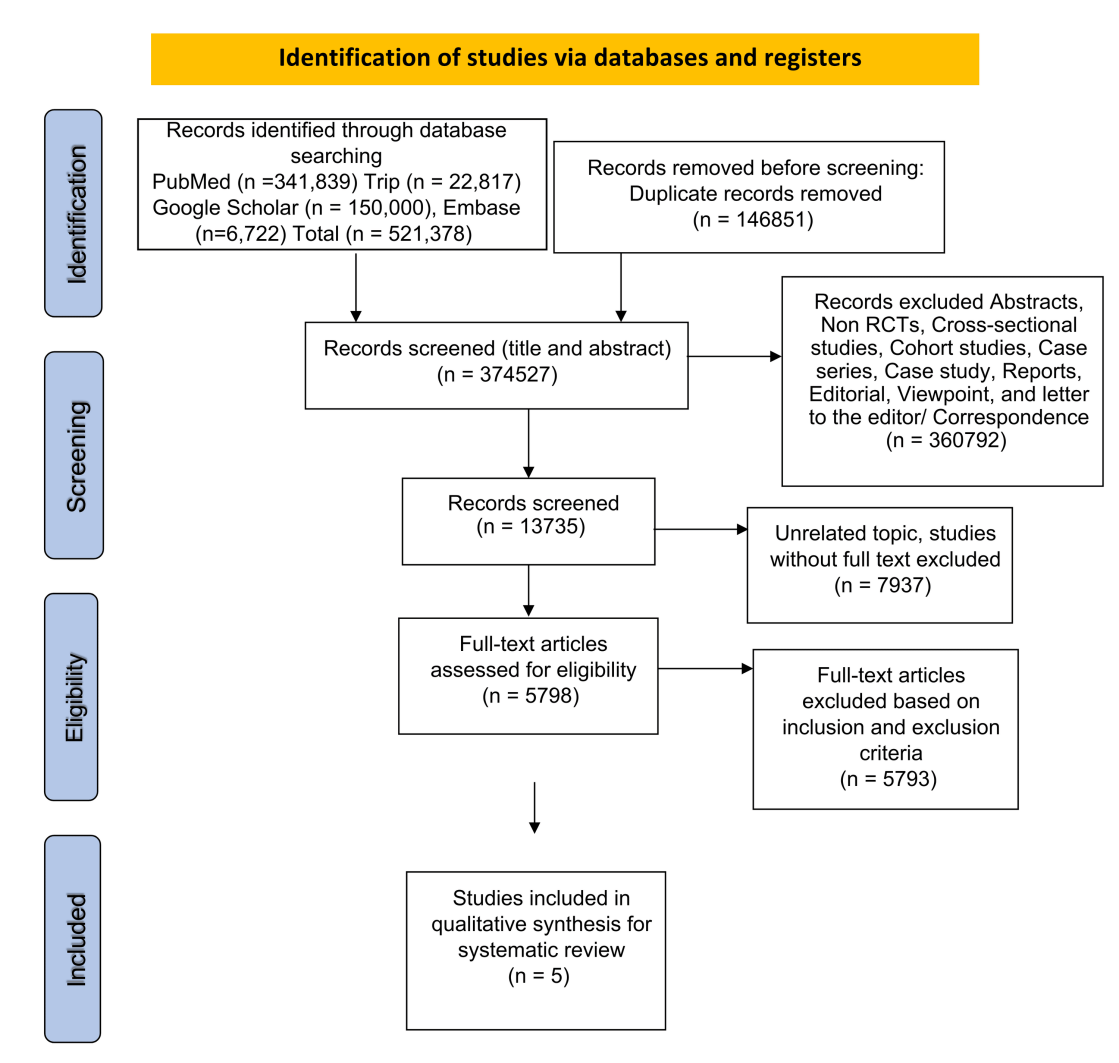
PRISMA 2020 flow diagram PRISMA: Preferred Reporting Items for Systematic Reviews and Meta-Analyses, RCT: randomized controlled trials

The literature search conducted generated a total of 521,378 articles, of which 146,851 were flagged as duplicates, which were excluded in the initial phase of screening. After further evaluation and analysis of the remaining pertinent titles and abstracts, via the application of the inclusion and exclusion criteria, 5,798 articles were further precluded from the analysis and quality assessment. In the final analysis, five studies adequately assessed the relationship between the use of exogenous testosterone replacement therapy in prostate carcinoma and were ultimately included in this current systematic review (Figure 1).

The systematic review ultimately revealed a triad of findings: (1) exogenous testosterone replacement therapy is not contraindicated in men who have undergone definite treatment for their prostate carcinoma (prostatectomy, external beam radiation, and/or chemotherapy), (2) exogenous testosterone replacement therapy does not alter intraprostatic dehydroepiandrosterone (DHEA) levels and thus does not attenuate or catalyze any change in the prostatic milieu, and (3) castration-resistant prostate carcinoma does not undergo disease progression when treated using a high dose of testosterone replacement therapy. The use of testosterone is an invaluable tool in men who have become hypogonadal due to primary treatment of the focal prostatic lesion, and ultimately improves and bolsters patients’ quality of life in a plethora of ways, from maintaining patients’ libido, muscle tone, and energy level to improving their psychological well-being [12,13].

Table 1 depicts a summary of the studies, sample size, inclusion criteria, and exclusion criteria. Table 2 illustrates the number of intervention patients, number of control group patients, biochemical profile, baseline PSA level, testosterone level, intraprostatic DHEA, combination of drug/s, therapeutic protocol, findings, limitations of the study, and study outcomes in patients receiving testosterone replacement therapy.

**Table 1:** Summary of studies, sample size, inclusion criteria, and exclusion criteria PSA: prostate-specific antigen, TRT: testosterone replacement therapy, FinRSPC: Finnish Randomized Study of Screening for Prostate Cancer

Author and year	Duration	Design	Number of intervention patients	Control group of patients	Inclusion criteria	Exclusion criteria
Izumi et al. (2021) [14]	12 weeks	Randomized controlled trial	41	40	Of male gender, pathologically and/or cytologically diagnosed cancer with advanced or metastatic lesions	Less than 20 years in age, previous prostatic cancer, surgical procedures within the study period, severe infection, severe benign prostatic hyperplasia
Morris et al. (2009) [15]	12 weeks (median)	Randomized controlled trial (phase 1)	Cohort 1: 3, cohort 2: 3, cohort 3: 6	0	Greater than 18 years of age; male gender; have undergone chemical, surgical, or medical castration; not received chemotherapy for at least 4 weeks before therapy; have histologically confirmed prostate carcinoma	Patients with severe cardiovascular disease, spinal cord compression, deranged liver function, kidney dysfunction beyond the acceptable criteria
Thirumalai et al. (2016) [16]	12 weeks	Randomized, placebo-controlled trial (double-blinded)	Cohort 2: 10, cohort 3: 10, cohort 4: 10, cohort 5: 10, cohort 6: 10	Placebo: 10	Healthy eugonadal men	An existing pathological disease of the prostate, patients aged greater than 55 years and those aged less than 25 years, aberrant liver function, a known case of androgen abuse or history thereof, anemia
Szmulewitz et al. (2009) [17]	Variable	Randomized controlled trial (phase 1)	Cohort 1: 6, cohort 2: 6, cohort 3: 6	0	Greater than 18 years of age, male gender, minimum absolute PSA value of 3 ng/mL, castration-resistant prostate carcinoma	An increase in the PSA greater than 3 times, a patient unable to tolerate TRT, advanced metastatic disease, uncontrolled comorbid conditions
Siltari et al. (2023) [18]	18 years	Randomized controlled trial	2,919 men on TRT	0	Of male gender aged between 55 and 67 years at baseline from the FinRSPC	Previous diagnosis of prostatic cancer, unrecorded/untraceable TRT use, patients lost to follow-up due to death or emigration

**Table 2:** Biochemical profile, combination of drugs, therapeutic protocol, findings, limitations, and outcome - indicates little sign of improvement in patient outcome after intervention. + indicates a greater sign of improvement in patient outcome after intervention. PSA: prostate-specific antigen, DHT: dihydrotestosterone, TRT: testosterone replacement therapy, GnRH: gonadotropin-releasing hormone, DDD: defined daily doses

Author and year	Biochemical profile: PSA level (ng/dL), PSA change (%), DHEA (ng/g), DHT (ng/dL)	Combination of drug/s	Therapeutic protocol	Main findings	Potential limitations	Outcome of therapy +/-
Izumi et al. (2021) [14]	Control: (mean and median) PSA at 8 weeks: +- 150, PSA at 12 weeks: +- 180; treated: PSA at 8 weeks: +- 120, PSA at 12 weeks: +-150	Testosterone enanthate	250 mg, intramuscular injection, 4 weekly	No overall survival difference was noted between the control and treated groups. General psychological improvement was found with the treatment group; however, no difference in physical parameters were noted.	Size of the study population, end points not specific to the progression of prostate carcinoma	+/-
Morris et al. (2009) [15]	Cohort 1: PSA change %: 5.48, DHT: 61.83; cohort 2: PSA change %: 11.4%, DHT: 63.2; cohort 3: PSA change %: -3.28, DHT: 102.31	Testosterone patch 5 mg, testosterone gel 1%	Testosterone patch 5%: cohort 1 (1 week) and 2 (1 month), testosterone gel 1%: cohort 3 (until disease progression), three times the recommended dose used	Castration-resistant prostate carcinoma does not undergo disease progression when treated using a high-dose testosterone replacement therapy	Size of the study population	+
Thirumalai et al. (2016) [16]	Cohort 1: PSA: 0.82, serum DHT: 36, intraprostatic DHEA: 29.3; cohort 2: PSA: 0.48, serum DHT: 37, intraprostatic DHEA: 26.2; cohort 3: PSA: 0.61, serum DHT: 43, intraprostatic DHEA: 22.1; cohort 4: PSA: 0.58, serum DHT: 39, intraprostatic DHEA: 26.8; cohort 5: PSA: 0.52, serum DHT: 49, intraprostatic DHEA: 24.4; cohort 6: PSA: 0.76, serum DHT: 36, intraprostatic DHEA: 18.6	GnRH antagonist (acyline), daily 1% transdermal testosterone gel	Cohort 1: placebo injections 2 weekly and placebo gel, cohort 2-6: GnRH antagonist (acyline) 300 μg/kg every 14 days, cohort 2: 1.25 g, cohort 3: 2.5 g, cohort 4: 5 g, cohort 5: 10 g, cohort 6: 15 g	Intraprostatic androgen concentrations do not have a linear relationship with serum androgen concentrations. No increase in PSA levels across any of the subjects.	Duration of the study and study population, lack of individuals with prostate carcinoma to monitor progression or regression thereof	+
Szmulewitz et al. (2009) [17]	Cohort 1: PSA: 840, cohort 2: PSA: 1,430, cohort 3: PSA: 1,320	Androderm® transdermal testosterone	Cohort 1: 2.5 mg/day, cohort 2: 5 mg/day, cohort 3: 7.5 mg/day	There was no statistically significant relationship between dose or testosterone level and time to progression using a Cox proportional hazards model (p=0.072 and p=0.14, respectively). Three patients demonstrated a decrease in PSA level during treatment.	Small study sample of only 12 subjects, non-placebo-controlled	+
Siltari et al. (2023) [18]	A total of 78,615 men, 2,919 of whom had received TRT, 9,265 of the total developed prostatic cancer, and 285 of those were treated with TRT	All oral injectable transdermal forms of TRT	No specific protocol, exposure to TRT was grounds for inclusion; standardized cumulative doses of TRT use were calculated based on DDD	Men using TRT were not associated with increased risk for prostate carcinoma and did not experience increased prostate carcinoma or cardiovascular disease-specific mortality compared to non-users.	-	+

**Figure 2:**
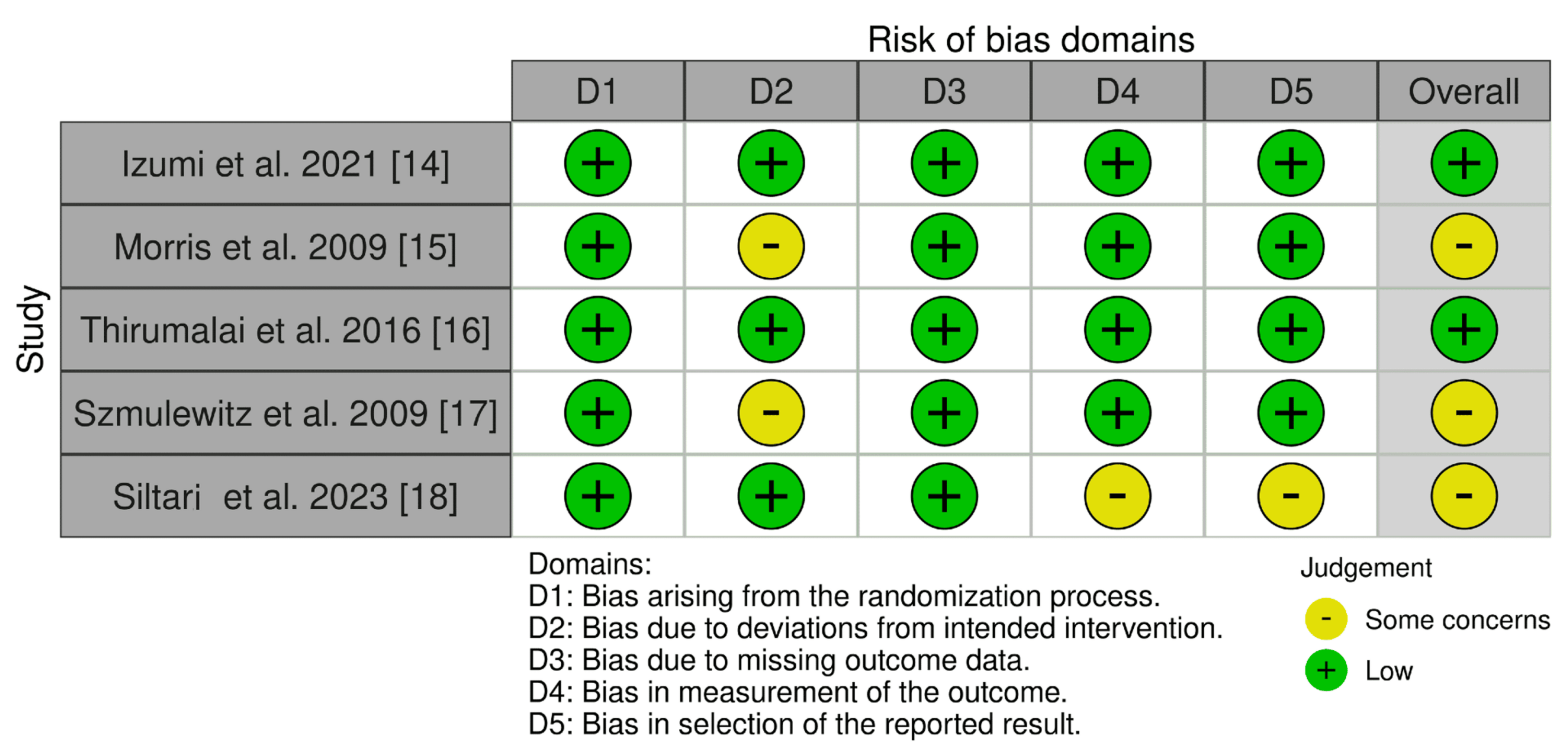
Traffic light plot of the RCTs (risk of bias assessment) RCT: randomized controlled trials

**Figure 3:**
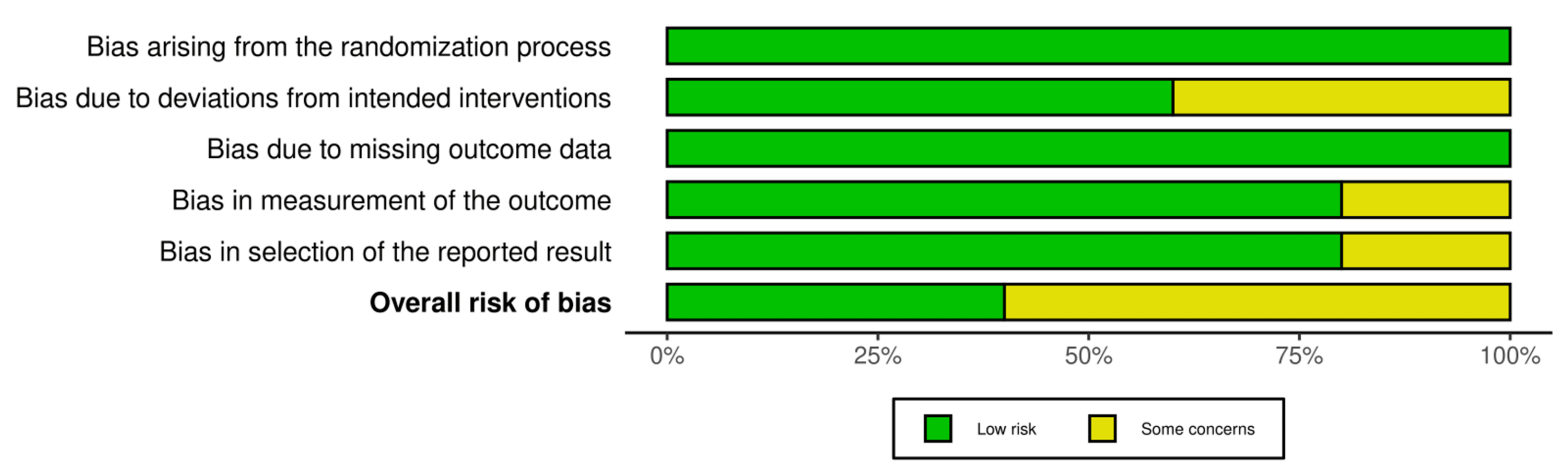
Weighted bar plot (risk of bias assessment)

Figures 2 and Figure 3 depict the risk of bias in all the included RCTs. Five domains were considered, which were as follows: bias arising from the randomization process (100% low risk), bias due to deviations from intended intervention (62.5% low risk), bias due to missing outcome data (100% low risk), bias in outcome measurement (25% some concerns), and bias in selection of reported result (25% some concerns). The robvis tool was used to generate the traffic light plot (Figure 2) and summary/weighted bar plot (Figure 3), which depicts the risk of bias.

Discussion

The systematic review ultimately revealed a triad of findings: (1) exogenous testosterone replacement therapy is not contraindicated in men who have undergone definite treatment for their prostate carcinoma (prostatectomy, external beam radiation, and/or chemotherapy), (2) exogenous testosterone replacement therapy does not alter intraprostatic dehydroepiandrosterone (DHEA) levels and thus does not attenuate or catalyze any change in the prostatic milieu, and (3) castration-resistant prostate carcinoma does not undergo disease progression when treated using a high dose of testosterone replacement therapy. The use of testosterone is an invaluable tool in men who have become hypogonadal due to primary treatment of the focal prostatic lesion, and ultimately improves and bolsters patients’ quality of life in a plethora of ways, from maintaining patients’ libido, muscle tone, and energy level to improving their psychological well-being [12,13]. No overall survival difference was noted between the control and treated groups, nor was there a difference in the physical parameters tested; however, general psychological improvements were noted in the treatment group in the randomized controlled trial undertaken by Izumi et al. (2021) on 41 intervention patients and 40 control group patients. The biochemical analysis of the study participants revealed the following (mean and median) PSA levels: PSA at eight weeks of +- 150 and at 12 weeks of +- 180 in the control cohort and PSA at eight weeks of +- 120 and at 12 weeks of +-150 in the treated cohort [14].

Morris et al. conducted a randomized phase 1 control trial with 12 intervention patients [15]. The inclusion criteria of the trial included only men greater than 18 years of age who had undergone chemical, surgical, or medical castration, not received chemotherapy for at least four weeks before therapy, and had a histologically confirmed prostate carcinoma. The hormonal therapy implemented in the first cohort was a 5 mg testosterone patch for one week, the second cohort were treated with a 5 mg testosterone patch for one month, and the third cohort were treated with testosterone gel 1% until natural disease progression. The relevant mean biochemical PSA change percentages and serum DHT levels in ng/dL in the three cohorts were reported as follows: PSA change percentage of 5.48 and serum DHT levels of 61.83 in cohort 1, PSA change percentage of 11.4% and serum DHT levels of 63.2 in cohort 2, and PSA change percentage of -3.28 and serum DHT levels of 102.31 in cohort 3. The PSA level decreased by 3.28% in the third cohort that received the TRT for the greatest duration, despite the serum DHT level being 102.31 ng/dL (near double that of the first two cohorts). This study proves that there is no correlation between increased serum testosterone levels and PSA levels, thereby proving that castration-resistant prostate carcinoma does not undergo disease progression when treated using a high-dose testosterone replacement therapy [15].

Exogenous TRT was proven to have no effect on the intraprostatic level of androgens in a randomized, placebo-controlled trial (double-blinded) study conducted by Thirumalai et al. (2016) on 50 interventional patients and 10 placebo patients [16]. The inclusion criteria in this study varied greatly from those in the other studies included in this systematic review, as healthy eugonadal men greater than 18 years of age were included in this study. All of the study participants were rendered to have iatrogenic hypogonadism via the administration of the gonadotropin-releasing hormone (GnRH) antagonist (acyline) 300 μg/kg every 14 days. The interventional cohort 2-6 were then treated with titrated doses of daily 1% transdermal testosterone gel and injections in the following doses: cohort 2, 1.25 g; cohort 3, 2.5 g; cohort 4, 5 g; cohort 5, 10 g; and cohort 6, 15 g. The high doses of TRT were up to three times the upper limit recommended for supplementation and replacement. The biochemical profile of the patients were reported as follows: PSA of 0.82, serum DHT of 36, and intraprostatic DHEA of 29.3 in cohort 1, PSA of 0.48, serum DHT of 37, and intraprostatic DHEA of 26.2 in cohort 2, PSA of 0.61, serum DHT of 43, and intraprostatic DHEA of 22.1 in cohort 3, PSA of 0.58, serum DHT of 39, and intraprostatic DHEA of 26.8 in cohort 4, PSA of 0.52, serum DHT of 49, and intraprostatic DHEA of 24.4 in cohort 5, and PSA of 0.76, serum DHT of 36, and intraprostatic DHEA of 18.6 in cohort 6. The ultimate conclusion from this study was that intraprostatic androgen concentrations do not have a linear relationship with serum androgen concentrations, and serum testosterone levels have no correlation or implication on the internal intraprostatic levels; thereby, high serum testosterone levels have no bearing on altering the internal prostatic milieu. Superadded to this, in line with and in support of both the PSA findings in Morris et al. [15] and Izumi et al. [14], no increase in PSA levels across any of the subjects despite the elevated serum testosterone was noted [16].

A randomized controlled trial (phase 1) study was undertaken by Szmulewitz et al. (2009) on 18 participants, with the following inclusion criteria: subjects greater than 18 years of age, male gender, a minimum absolute PSA value of 3 ng/mL, and castration-resistant prostate carcinoma [17]. The participants were divided into three cohorts and were treated with Androderm transdermal testosterone in the respective doses: cohort 1, 2.5 mg/day; cohort 2, 5 mg/day; and cohort 3, 7.5 mg/day. The study found that there was no statistically significant relationship between dose or testosterone level and time to progression using a Cox proportional hazards model (p=0.072 and p=0.14, respectively). It was also evident that despite the testosterone therapy, three of the patients recorded a decrease in PSA levels, which again corroborates and further supports previous findings [17].

A randomized controlled trial conducted by Siltari et al. comprised a total of 78,615 men, 2,919 of whom had received TRT, 9,265 of the total developed prostatic cancer, and 285 of those were treated with TRT. Men using TRT were not associated with increased risk for prostate cancer and did not experience increased prostate cancer or cardiovascular disease-specific mortality compared to non-users [18].

A randomized controlled trial was undertaken by Izumi et al. (2021) on 41 intervention patients and 40 control group patients. The inclusion criteria of participants were male gender and pathologically and/or cytologically diagnosed cancer with advanced or metastatic lesions. The trial lasted a median of 12 weeks. The therapeutic protocol implemented in the trial was a 250 mg intramuscular injection of testosterone enanthate four weekly. The trial did not track progression of the cancer as a primary end point, but more so tracked cancer symptoms and hypogonadal features in those with and without the TRT to conclude whether TRT in hypogonadal men following primary treatment for prostate carcinoma is viable and useful. The biochemical profiles of the study participants were reminiscent of those seen in the study undertaken by Morris et al. [15], whereby PSA levels were relatively unchanged despite exogenous TRT. Ultimately, no overall survival difference was noted between the control and treated groups. A general psychological improvement was found with the treatment group; however, no difference in physical parameters were noted. The limitations of the study are the size of the study population and its end points not being specific to the progression of prostate carcinoma [14]. This study is both supported and contradicted by a study undertaken by Ramasamy et al. [19], who concluded, parallel to Izumi et al. [14], that no correlation between prostate carcinoma progression and TRT was evident. However, in contradiction to Izumi et al. [14], Ramasamy et al. [19] noted the benefits of TRT in hypogonadal men following their primary treatment of prostate carcinoma. The benefits of TRT include bolstering patients’ quality of life in a plethora of ways, from maintaining the patients’ libido and muscle tone, to preventing osteoporosis, to improving energy levels, to improving their psychological well-being [19].

The importance of this study is that it correlates and is further supported by a systematic review undertaken by Kardoust Parizi et al., who concluded that no elevated rates of prostate carcinoma progression were present after TRT in patients following their definitive therapy [20]. The study further supports the notions of Izumi et al. [14], where TRT is indicated in hypogonadal men for its myriad of benefits.

The use of TRT is not contraindicated in men who have received definitive therapy for their primary neoplastic prostatic lesion, and it should be initiated in those men who suffer from hypogonadism secondary to the treatment. The use of testosterone therapy in untreated or high-risk men is still debatable, as various animal studies have shown that testosterone does cause prostate carcinoma progression in rats [21].

Limitations of the Study

Due to the small number and heterogeneity of the included RCTs, a meta-analysis or statistical synthesis was not performed. The review presents a qualitative summary without pooled estimates or confidence intervals. This study is limited in terms of the wide array and types of testosterone replacement therapies that were included among the various randomized controlled trials that form part of this systematic review. This is unfortunate, as not a single type of replacement can be compared to another using baselines. The effects of various testosterone formulations on the prostate and the progression of the cancer can thus not be accurately compared between the various formulations.

Future Directions

The role of TRT in prostate cancer is evolving, even in cases of advanced prostate cancer. One area of interest is bipolar androgen therapy (BAT), which has been studied in various clinical trials focused on castrate-resistant prostate cancer (CRPC). Prostate cancer cells adapt to chronic low testosterone levels by increasing androgen receptor activity, resulting in a 30- to 90-fold rise in androgen receptor levels. Although this significant upregulation of androgen receptors can lead to castration resistance, it also creates a therapeutic vulnerability to treatment with high doses of testosterone, which can result in growth arrest or cell death. The term “bipolar” in BAT refers to the rapid switching between two extremes: from high testosterone levels (supraphysiologic) to near-castrate serum testosterone levels. This cycling can be achieved by administering testosterone cypionate at the Food and Drug Administration (FDA)-approved dose of 400 mg intramuscularly once every 28 days while simultaneously maintaining continuous testosterone suppression through surgical castration or the use of luteinizing hormone-releasing hormone (LHRH) agonists or antagonists [22-29].

## Conclusions

No correlation between testosterone replacement therapy and prostate carcinoma exists, and TRT is not contraindicated in men post-definitive treatment for their primary neoplastic prostatic lesion. TRT is indicated in hypogonadal men post-primary treatment for its myriad of benefits and ultimate improvement of quality of life. Although preliminary data are promising, high-dose testosterone should still be considered experimental and only administered within clinical trial settings. The role of TRT in men prior to the diagnosis of prostate carcinoma is unclear, and thus, patients should give their informed consent before receiving the testosterone therapy.
